# Comparative Studies of Different Ablation Techniques for Atrial Fibrillation

**DOI:** 10.31083/RCM33490

**Published:** 2025-05-20

**Authors:** Monika Keževičiūtė, Germanas Marinskis, Diana Sudavičienė, Jūratė Barysienė, Neringa Bileišienė, Greta Radauskaitė, Audrius Aidietis, Gediminas Račkauskas

**Affiliations:** ^1^Department of Cardiovascular Medicine, Faculty of Medicine, Vilnius University, LT-0310 Vilnius, Lithuania; ^2^Center of Cardiology and Angiology, Vilnius University Hospital Santaros Clinics, LT-08406 Vilnius, Lithuania

**Keywords:** atrial fibrillation, radiofrequency ablation, cryoablatiobn, pulsed fieldablation, pulmonary vein isolation

## Abstract

Atrial fibrillation (AF) is the most common supraventricular arrhythmia, affecting 2–3% of the adult population, with an increasing prevalence due to demographic shifts; however, detection methods have also improved. This rhythm disorder is associated with significant morbidity, manifesting through symptoms that worsen the quality of life, as well as with adverse outcomes and increased mortality. The substantial AF burden on the healthcare system necessitates the development of effective and durable treatment strategies. While pharmacological management represents the first-line approach for AF, the limitations associated with this approach, including side effects and insufficient efficacy, have promoted the evolution of catheter ablation techniques that isolate pulmonary veins (PVs) and, thus, disrupt arrhythmia-causing impulses from the atria. Currently, three energy sources have gained U.S. Food and Drug Administration (FDA) and European regulatory approval (The Conformité Européene (CE) mark certification) for catheter ablation: radiofrequency ablation (RFA), cryoballoon ablation (CBA), and, more recently, pulsed-field ablation (PFA). RFA has subsequently become an effective treatment, demonstrating superior outcomes in randomized controlled trials compared to antiarrhythmic drug therapy. CBA has also proven to be a safe and effective alternative, particularly for patients with symptomatic paroxysmal AF, showing comparable efficacy to RFA and similar rates of complications. Meanwhile, PFA is emerging as a promising technique, offering non-inferior efficacy to conventional thermal methods while potentially minimizing the thermal damage to adjacent tissues associated with RFA and CBA. Despite higher equipment costs, the advantages of PFA in reducing complications highlight its potential role in AF management. However, considering the novelty of PFA, no data currently exist comparing this strategy with thermal techniques. Therefore, further research is needed to improve the management of AF and patient outcomes to reduce healthcare burdens.

## 1. Introduction

Atrial fibrillation (AF) is the most common supraventricular arrhythmia, characterized by uncoordinated atrial electrical activation and ineffective atrial contraction. It affects 2–3% of the adult population, and its prevalence is expected to double by 2060 due to an aging population and improved detection [1]. This rhythm disorder is manifested through a variety of symptoms such as palpitations, shortness of breath, fatigue, and reduced exercise capacity, and is associated with adverse outcomes, including ischemic events, recurrent hospitalizations, heart failure, cognitive decline, impaired quality of life, and increased mortality [2, 3].

AF has resulted in an increasing burden on society and healthcare systems. Its increasing prevalence leads to greater economic costs and overloads healthcare services [4]. These challenges necessitate the development of more effective, safe, and durable treatment strategies.

Currently, the first-line treatment for AF is pharmacological, however, it may be associated with various side effects and may not always be effective. In 1987 James Cox introduced the Cox-maze procedure, in which multiple incisions are created in both the left and the right atria to eliminate AF while allowing the sinus node impulse to reach the atrioventricular node. However, this procedure and its modifications necessitate open surgical approaches [5].

Since the electrical activity in the pulmonary veins (PVs) was first described as a primary trigger for AF in 1998, catheter ablation techniques have been developed to disrupt the electrical connections between the PV and the left atrium, offering a less invasive alternative to traditional surgical methods [6].

Today, PV isolation can be achieved by various percutaneous technologies that use different energy sources. To date, three energy sources have gained Food and Drug Administration (FDA) and Conformité Européene (CE) mark approval for this purpose: radiofrequency ablation (RFA), cryoballoon ablation (CBA) and more recently, pulsed field ablation (PFA).

The aim of this article is to review the current studies that compare various ablation techniques for AF, highlighting their differences in safety and effectiveness, as well as to identify areas where data is still lacking. In this article standard energy sources, as RFA and CBA are compared with antiarrhythmic drug therapy and compared with each other. Available data comparing PFA with thermal ablation technologies is presented as well. Additional aspects, such as anesthesia or parameters of catheters are briefly summarized in the end.

## 2. Radiofrequency Ablation vs Antiarrhythmic Drug Therapy

The first energy source introduced in clinical practice was radiofrequency. This energy source is still widely used, and its mechanism of action is based on coagulative necrosis of tissue from increased temperatures [7]. There are several randomized controlled trials (RCTs) comparing effectiveness of RFA to antiarrhythmic drug therapy (ADT) in managing paroxysmal AF. RFA superiority was documented in patients with paroxysmal AF who had not responded to at least one antiarrhythmic drug, showing a longer time to recurrent arrhythmias [8]. Another RCT by Cosedis Nielsen *et al*. [9] confirmed the durable benefits of RFA, showing that over a 24-month period, the AF burden, measured as the percentage of time spent in AF during Holter monitoring, was significantly lower in the RFA group than in the ADT group. These findings emphasize RFA’s sustained efficacy in reducing AF episodes and improving long-term outcomes. Another RCT by Mont *et al*. [10] on persistent AF shows an advantage of RFA compared to ADT at 12-month follow-up. RFA has demonstrated better results as compared to ADT in treatment with symptomatic paroxysmal AF [11]. These results were supported by another RCT by Wazni *et al*. [12], where patients with both paroxysmal and persistent AF and without previous ADT were included, suggesting that RFA can be a feasible first-line approach. Detailed findings of all these RCTs are summarized in Table 1 (Ref. [8, 9, 10, 11, 12]).

**Table 1.** **RFA vs ADT**.

Study, year, reference	Design, number of patients	Clinical characterization of AF	Follow-up period	AF detection method	RFA superior to AF (Yes/no, *p* value)	Adverse events, mortality
The Thermocool-AF trial, 2009 [8]	A prospective multicenter randomized unblinded study, N = 167 (RFA N = 106, ADT N = 61)	Paroxysmal	9 months	ECG at FU visits, Transtelephonic monitoring, Holter monitoring	Yes, *p* < 0.001	30-day major treatment-related adverse events occurred in 5 patients (1 pericardial effusion, 1 pulmonary edema, 1 pneumonia, 1 vascular complication, and 1 heart failure) in the catheter ablation group (5/103 [4.9%]) and 5 patients (2 with life-threatening arrhythmias and 3 with disabling drug intolerance requiring discontinuation) in the ADT group (5/57 [8.8%]). One death in RFA group 284 days after the procedure due to acute myocardial infarction (unrelated to the procedure).
Wazni *et al*., 2001–2002 [12]	Multicenter prospective randomized study, N = 70 (RFA N = 33, ADT N = 37)	Paroxysmal and persistent	1 year	loop event-recorder, Holter monitoring	Yes, *p* < 0.001	No thromboembolic events in either group. Bleeding rates were similar. Bradycardia was higher in the antiarrhythmic drug group (3 [8.6%] of 35 patients vs none in the PVI group). Asymptomatic moderate (50%–70%) pulmonary vein stenosis was documented in 1 (3%) of 32 patients in the PVI group affecting only 1 vein; no patient developed severe (>70%) pulmonary vein stenosis.
MANTRA-PAF Trial, 2012 [9]	Multicenter, randomized trial, N = 294 (RFA N = 146, ADT N = 148)	Paroxysmal	2 years	7-day Holter monitoring	Yes, *p* = 0.007	20 patients in the RFA and 16 patients in ADT group had serious adverse events (*p* = 0.45). In RFA group 3 patients had cardiac tamponade. 3 patients in the RFA and 4 patients in ADT group died during the study. One death in the RFA group was caused by a procedure-related cerebral stroke. The other causes of death were not considered to be related to the treatment.
RAAFT-2 trial, 2006–2012 [11]	Randomized clinical trial, N = 127 (RFA N = 61, ADT N = 61)	Paroxysmal	2 years	Scheduled or unscheduled ECG, Holter monitoring, transtelephonic monitor, rhythm strip	Yes, *p* = 0.02	RFA group had a 9% rate of serious adverse events, the most frequent of which was pericardial effusion with tamponade experienced by 4 patients (6.0%). 1 severe pulmonary vein stenosis, bradycardia leading to pacemaker insertion in RFA group. No deaths.
SARA trial, 2009–2013 [10]	Multicentre randomized trial, N = 146 (RFA N = 98, ADT N = 48)	Persistent	1 year	ECG, Holter monitor	Yes, *p* < 0.001	In the RFA group 6 patients (6.1%) had periprocedural complications: 2 pericarditis, 1 pericardial effusion and 3 minor vascular access complications that did not require intervention. During follow-up, 1 patient under oral anticoagulation had spontaneous renal hematoma and 1 patient had symptomatic pulmonary vein stenosis requiring stenting. The ADT group had one flecainide intoxication and one minor vascular access complication (4.2% of patients). No deaths, transient ischemic events, or strokes were documented in either group.

AF, atrial fibrillation; RFA, radiofrequency ablation; ADT, antiarrhythmic drug therapy; FU, follow-up; N, number; PVI, pulmonary vein isolation; ECG, electrocardiogram.

Regarding adverse events, most complications in RFA procedures typically result in acute morbidity rather than long-term consequences [12]. These complications often are associated with vascular access, such as femoral pseudo-aneurysms, arteriovenous fistulas or bleeding complications. They are mostly considered minor and do not require intervention [10]. Other less common adverse events include pericardial effusion, cardiac tamponade, and asymptomatic or symptomatic PV stenosis [11]. Only one death in the RFA group was reported because of a procedure-related cerebral stroke [9]. A relatively rare, although one of the most feared complications is atrioesophageal fistula, which is associated with amortality of 70–80% and is generally fatal if not recognized early and treated surgically [13].

## 3. Cryoballoon Ablation vs Antiarrhythmic Drug Therapy

Catheter balloon cryoablation, in which evaporating nitrous oxide gas decreases temperature to –40–50 °C and freezes target tissue to cause necrosis, is the second energy source widely used in clinical practice. Since 2012, the second-generation cryoballoon is used in clinical practice [14]. This method was quickly adopted among electrophysiology centers, based on evidence suggesting that second-generation CBA demonstrates excellent learning curves for new operators [15]. A large RCT proved CBA was a safe and effective alternative to ADT for patients with highly symptomatic paroxysmal or persistent AF and who experienced failure of at least one antiarrhythmic drug [16]. Two other RCTs further confirmed the superiority of CBA as an initial therapy for preventing the recurrence of trial arrhythmias in patients with paroxysmal AF [17, 18]. Additional data was provided from the EARLY-AF trial which included continuous cardiac rhythm monitoring. This RCT further reaffirmed cryoablation as an initial treatment for symptomatic paroxysmal AF, with a significantly lower rate of AF recurrence in the cryoballoon ablation group compared to the antiarrhythmic drug group [19]. Significant findings supporting the use of ablation as a strategy to prevent disease progression in patients with paroxysmal AF were obtained in a 3-year follow-up, where catheter cryoballoon ablation was associated with a lower incidence of persistent AF or recurrent atrial tachyarrhythmia compared to initial treatment with antiarrhythmic drugs [20]. Detailed findings of all these RCTs are summarized in Table 2 (Ref. [16, 17, 18, 19, 20]).

**Table 2.** **CBA vs ADT**.

Study, year, reference	Design, number of patients	Clinical characterization of AF	Follow-up period	AF detection method	CBA superior to AF (Yes/no, *p* value)	Adverse events, mortality
STOP AF pivotal trial, 2013 Packer [16]	Prospective, multicenter, randomized, controlled study	Paroxysmal and persistent, N = 245 (CBA N = 163, ADT N = 82)	1 year	Personal trans-telephonic monitoring, Holter monitoring	Yes, *p* < 0.001	Five CBA patients experienced major AF events: 1 (0.6%) patient sustained an unrelated fatal MI at 10 months; 1 patient had Wegener’s-related hemoptysis, AF recurrence and hospitalization for antiarrhythmic drug adjustment; 1 patient had a subarachnoid hemorrhage; 1 patient had intestinal bleeding accompanying an elevated INR; and 1 patient was hospitalized with AF-related congestive heart failure.
STOP AF First, 2021 [17]	Multicenter trial	Paroxysmal	1 year	ECG, patient-activated telephone monitoring, Holter monitoring	Yes, *p* < 0.001	A serious adverse event occurred in 14% of the patients in the ablation group and in 14% of the patients in the drug-therapy group. There were no cases of pulmonary vein stenosis.
		Naive N = 203 (CBA N = 99, ADT N = 104)				
Cryo-FIRST, 2021 [18]	Multicentre, prospective, open blind-endpoint controlled randomized study	Paroxysmal	1 year	ECG, 7-day Holter monitoring, a patient diary	Yes, *p* = 0.01	No occurrences of death, atrio-esophageal fistula, stroke, pericardial tamponade, or chronic phrenic nerve injury within the CBA cohort.
		Naive N = 220 (CBA N = 154, ADT N = 149)				
EARLY-AF, 2021 [19]	Investigator-initiated, multicenter, open-label, randomized trial with blinded end-point adjudication	Paroxysmal	1 year	Implantable cardiac monitoring device	Yes, *p* < 0.001	There were no procedural deaths or thromboembolic complications, the most common periprocedural complication was self-limited phrenic nerve palsy.
		Naive N = 303 (CBA N = 107, ADT N = 111)				
EARLY-AF 3 years FU, 2022 [20]	Investigator-initiated, multicenter, open-label, randomized trial with blinded end-point adjudication	Paroxysmal	3 years	Implantable cardiac monitoring device	Yes, *p* < 0.001	At 36 months of follow-up, adverse events had occurred in 17 patients (11.0%) in the ablation group and in 35 patients (23.5%) in the antiarrhythmic drug group. In the ablation group, these adverse events included one death, three cases of phrenic nerve palsy that resolved spontaneously, and two pacemaker implantations.
		Naive N = 303 (CBA N = 107, ADT N = 111)				

CBA, cryoballoon ablation; MI, myocardial infarction; INR, International Normalized Ratio.

CBA demonstrated a safe profile in most of the trials. This method showed a similar rate of serious adverse events between both treatment arms (CBA and ADT) [18]. The EARLY-AF trial further highlighted the safety of cryoablation, reporting fewer serious adverse events in the ablation group compared to the ADT group (3.2 vs 4%) [21]. The most common periprocedural complication was transient phrenic nerve palsy [20].

## 4. Radiofrequency Ablation vs Cryoballoon Ablation

Animal studies have shown a higher risk of thrombus formation after RFA than after CBA. Two studies have compared cryoenergy and radiofrequency (RF) energy in terms of their effects on coagulation, inflammation, and myocardial tissue destruction. Although both studies failed to prove CBA has a safer procedure profile compared to RFA, despite the greater myocardial injury in RFA, the markers of coagulation, endothelial damage, and inflammation were similar between the two techniques [22, 23]. Another study, AF-COR, compared the efficacy, safety and procedure times of these two energy sources [24]. Both techniques demonstrated similar effectiveness and safety in achieving acute pulmonary vein isolation (PVI), although CBA had had significantly shorter fluoroscopy times. Patients were followed up for 1 year, and significant improvement of arrhythmia-related symptoms and quality of live were similar in both groups after ablation [24]. However, when comparing these two energy sources in patients undergoing a redo procedure when a previous first RFA failed, RFA may be a preferable ablation strategy as it was associated with a better AF-free outcome [25]. Another RCT introduced a novel combined strategy, where RFA was followed CBA, and compared it with standard RFA and CBA alone [26]. Both the combined approach and CBA were superior to conventional RFA. Although the combined strategy was not significantly better than CBA alone, the CBA group showed higher single procedure success compared to RFA. Moreover, the results were consistent with previous findings, showing shorter fluoroscopy times with cryoballoon ablation [26]. In 2016, the FIRE AND ICE trial demonstrated that CBA was non-inferior to RFA in terms of both efficacy and safety for symptomatic patients with paroxysmal AF [27]. However, analysis of secondary endpoints revealed significant advantages for cryoballoon ablation, as patients treated with cryoballoon required fewer repeat ablations, fewer cardioversions, and experienced fewer all-cause and cardiovascular-related rehospitalizations during follow-up [27]. Similar results were demonstrated in the Freeze AF trial, where CBA was compared to RFA in patients with drug-refractory paroxysmal AF [28]. Freedom from AF without persistent complications over the 30-month follow-up was evaluated and success rates of both groups were similar, demonstrating CBA to be non-inferior to RFA [28]. Second-generation cryoballoon (CB) demonstrated more durable PV isolation, as well as improved freedom of atrial tachyarrhythmias in comparison with RFA in patients with drug-refractory paroxysmal AF in the study by Buist *et al*. [29]. Notably, in this study, acute PV isolation was always achieved using both ablation strategies. However, another trial comparing second generation cryoballon vs RFA provided similar data on early recurrence rates of AF but emphasizes its earlier occurrence in the initial phase after RFA ablation when compared with CBA [30]. As none of the previous comparative studies have found significant differences in complication rates, the most often reported complication in the RFA group was PV stenosis due to thermal damage. Another study was conducted to investigate whether there was any difference in the extent of acute or chronic PV stenosis after PVI between the two energy sources [31]. While no significant difference was observed between the groups 3 months post-ablation, the authors suggested that CBA may reduce acute stenosis of the left-sided PV compared to RFA [31]. Another study evaluating the efficacy, safety, and procedural profiles of AF ablation technologies was CIRCA-DOSE [32]. This study was the first comprehensive evaluation of spontaneous and provoked PV reconnection. Patients were assessed after a 20-minute observation period and again following provocative testing with adenosine. This study provided strong evidence that patients with acute intraprocedural PV reconnection, even if eliminated through additional ablation, experienced significantly worse long-term freedom from recurrent AF. Acute PV reconnections, whether spontaneous or adenosine-provoked, were significantly more frequent in the RFA group. Furthermore, the patterns of affected PVs and the sites of reconnection varied depending on the ablation technology used. This study underscores the importance of achieving optimal ablation lesions during the initial procedure to ensure durable PV isolation and improve long-term outcomes [32]. Despite a higher incidence of PV reconnections in the RFA group, no significant differences were observed in health-related quality-of-life improvements or reductions in healthcare utilization during the year following the ablation procedure. This suggests that, although RFA may lead to more PV reconnections, both RFA and cryoballoon ablation provide similar benefits in terms of patient-reported outcomes and overall reduction of the healthcare burden [33]. While the common parameter to assess effectiveness of catheter ablation has traditionally been linked to absolute freedom from recurrent arrhythmia, a sub-study of the CIRCA-DOSE trial found that quality of life is associated with significant reductions in the frequency of arrhythmias and showed that AF burden significantly decreased at 12 months post-ablation [34]. These outcomes may also be linked to changes in autonomic function, as PV isolation, regardless of the ablation technology used, leads to sustained alterations in heart rate parameters. Specifically, patients often experience decreased heart rate variability along with increases in both daytime and nighttime heart rates [35]. These autonomic changes could contribute to the improvements in quality of life and reduction in arrhythmia burden observed after ablation.

Study of another design was carried out in 2019, in which patients with drug-refractory paroxysmal AF, were assigned to three groups: 4-minute cryoballoon ablation, 2-minute cryoballoon ablation, or contact force–guided radiofrequency ablation [21]. The patients were followed for one year, and a continuous cardiac rhythm monitoring was conducted. The study demonstrated that both radiofrequency ablation and the two different cryoballoon ablation protocols resulted in similar one-year efficacy according to the time to first recurrence of atrial arrhythmia. However, all treatment modalities showed a significant AF burden reduction of over 98%, as assessed by continuous cardiac rhythm monitoring. This study supported previous results, showing that the rate of arrhythmia recurrence may be comparable across techniques, but the overall reduction in AF burden remains highly effective, regardless of the ablation method used [21].

In patients with persistent or long-standing persistent AF, the NO-PERSAF study found no significant differences in effectiveness between CBA and RFA [36]. At the 12-month follow-up, both techniques showed similar rates of freedom from atrial tachyarrhythmias. However, the study found that patients in the CBA group had a lower recurrence of atrial flutter compared to the RFA group. Additionally, CBA was associated with shorter procedure and ablation times, offering a procedural advantage over RFA [36].

These findings were further confirmed by a 2023 study, in which patients with persistent AF were enrolled in a 2:1 ratio (RFA:CBA) [37]. The study demonstrated that both techniques were equally effective for rhythm control, further supporting the significant advantage of CBA in terms of shorter procedure duration [37].

However, a recently published sub-study of a CIRCA-DOSE trial, provided further insight on disease progression, as RFA was associated with less patients experiencing an episode of persistent atrial tachyarrhythmia, as determined by implantable cardiac monitors, compared with patients randomized to CBA [38]. This difference in progression was observed despite similar rates of arrhythmia recurrence, a similar low burden of AF during the early and late follow-up period, and a similar profound reduction in AF burden from baseline [38].

In contrast, when considering PV antral scar on post-ablation cardiac magnetic resonance, CBA demonstrated greater percentage compared to RFA, suggesting more effective scar location [39].

Both techniques did not differ in safety and had a very low rate of complications. The most common complications in both groups were phrenic nerve injury resulting in diaphragmatic paralysis, PV stenosis, or more dangerously, damage to the esophagus, which can result in an atrial-esophageal fistula, all of them due to thermal injury on structures adjacent to the PV [40].

No randomized trial has directly compared modern RFA techniques, such as index-guided ablation or very high-power short-duration ablation, with CBA. While both modalities are effective for PV isolation, RFA offers greater procedural flexibility. Unlike CBA, which is primarily designed for circumferential PV isolation, RF ablation allows precise, point-by-point lesion creation. This flexibility enables electrophysiologists to modify their strategy intraoperatively and more easily target non-PV triggers, which may play a role in atrial fibrillation persistence or recurrence. This adaptability is particularly relevant for complex cases requiring additional substrate modification beyond standard PV isolation [41].

## 5. Pulsed Field Ablation vs Thermal Ablation

Recently, a novel technique known as PFA has been introduced into clinical practice. This nonthermal energy modality has the potential for deeper tissue penetration compared to traditional thermal ablation techniques, such as RFA or CBA. This is due to the unique mechanism of action of PFA, which utilizes high-voltage, short-duration electrical pulses to create irreversible electroporation in the targeted tissue [42]. Unlike thermal modalities, which rely on heat conduction to create lesions, electrical fields of PFA can affect a broader area of tissue, potentially allowing for greater penetration and more uniform lesion formation. As a result, PFA may achieve superior transmurality, ensuring that the lesions extend through the full thickness of the myocardial wall. This deeper and more consistent tissue effect could lead to more durable ablation results, especially in areas that are difficult to treat with traditional techniques, such as in thicker or fibrotic tissue regions, utilizes high-voltage electrical currents to irreversibly electroporate cardiac tissue [43]. In this non-thermal method, preclinical and clinical studies have shown that PFA has a similar potential to induce myocardial necrosis while minimizing the thermal impact on surrounding structures [44]. No signs of esophageal injury were reported after PFA using cardiac magnetic resonance imaging [45]. PFA for paroxysmal AF demonstrated a clinical success rate of 87.5%, with significant improvement in quality of life, reductions in the use of ADT, cardioversion, and hospitalization [46]. However, currently there is a lack of randomized clinical trials comparing PFA with traditional ablation techniques such as RFA or CBA. A meta-analysis of 11 studies comparing PFA with CBA demonstrated that PFA results in shorter procedure times, lower arrhythmia recurrence rates, and a reduced risk of periprocedural complications compared to CBA [47]. Only a few studies have compared PFA to thermal ablation, when RFA and CBA are studied together.

The ADVENT trial demonstrated that in patients with paroxysmal AF, PFA was noninferior to conventional thermal ablation [46]. PFA matched thermal ablation in terms of the primary efficacy endpoint—freedom from procedural failure, atrial tachyarrhythmia after a 3-month blanking period, use of antiarrhythmic drugs, cardioversion, or repeat ablation. PFA showed similar safety outcomes, with no significant differences in device and procedure related serious adverse events at the 1-year follow-up. In this study, no complications were related to the energy delivered in the PFA patients [48].

Recently a 30-second atrial arrythmia recurrence rate as a primary end point was criticized, as it lacks clinical significance and significantly underestimates the effectiveness of ablation therapies [49, 50]. Thus, the post-ablation atrial arrhythmia burden was suggested as a better parameter to evaluate outcomes [51]. Based on this endpoint, data from the ADVENT trial was re-analyzed and the results demonstrated better reduction of the 1-year post-ablation atrial arrhythmia burden with PFA compared to thermal ablation. The analysis also showed a significant improvement in atrial arrhythmia burden favoring PFA in patients resistant to AAD. These findings suggest that PFA may offer enhanced effectiveness over thermal ablation, based on this post hoc analysis of the randomized study [52]. There is data supporting PFA as a preferable method for redo procedures following a previous RFA [53].

PFA cardio-selectivity is considered as an advantage in reducing complications, and fibrotic proliferation which results in significantly less PV stenosis [54]. However, there is data showing that is has a lesser effect on the autonomic nervous system. Previous studies have demonstrated that this additional effect of thermal ablation contributes to enhanced symptom relief and improved long-term freedom from arrythmias [35, 55].

A recent meta-analysis involving 24 studies and 5203 patients compared the safety and acute efficacy of PFA with thermal ablation techniques [54]. The results indicated that PFA was associated with lower periprocedural complication rates, while achieving comparable rates of acute procedural success and similar recurrence of AF up to one-year post-procedure [56]. The most reported complication, coronary vasospasm, has been shown to be subclinical in most cases and effectively managed prophylactically or post hoc with nitroglycerin [57].

No studies have directly compared very high-power short-duration RFA with pulsed PFA. However, observational data suggest that both techniques may offer similar effective outcomes in terms of procedural success, complication rates, and long-term arrhythmia control.

No studies have directly compared very high-power short-duration RFA with pulsed PFA. However, observational data suggest that both techniques may offer similar effective outcomes in terms of procedural success, complication rates, and long-term arrhythmia control.

When comparing PFA to thermal ablation methods in terms of healthcare costs, PFA demonstrated advantages with shorter skin-to-skin and catheter laboratory times, as well as similarly low complication rates. However, PFA resulted in higher equipment costs, which may impact its overall cost-effectiveness [58]. On the other hand, a cost-effectiveness analysis of the ADVENT study demonstrated that PFA presents a viable, cost-effective alternative to thermal ablation, supporting its broader adoption in clinical practice. In this analysis PFA was superior to thermal ablation in terms of health outcomes and cost savings over a 40-year horizon. PFA was associated with fewer strokes, lower treatment failure rates, and increased health utility. Key uncertainty drivers included anticoagulation costs, procedure costs, and AF progression rates. Budget impact analysis suggested PFA is an affordable short-term option, with potential long-term financial benefits. Future research should focus on healthcare utilization, sedation protocols, and long-term transition rates to refine cost-effectiveness models [59]. Detailed findings of all these RCTs are summarized in Table 3 (Ref. [55, 59]).

**Table 3.** **PFA vs thermal ablation**.

Study, year, reference	Design, number of patients	Clinical characterization of AF	Follow-up period	AF detection method	Findings	Adverse events, mortality
ADVENT, 2023 [55]	randomized, single-blind, noninferiority trial	Paroxysmal	1 year	72-hour Holter monitoring, transtelephonic ECG recordings	Pulsed field ablation was noninferior to conventional thermal ablation	Device- or procedure-related serious adverse events (primary safety end point) occurred in 6 patients who underwent pulsed field ablation and 4 patients who underwent thermal ablation
ADVENT, 2024 [59]	randomized, single-blind, noninferiority trial	Paroxysmal	1 year	72-hour Holter monitoring, transtelephonic ECG recordings	Compared with thermal ablation, PFA more often resulted in an AA burden less than the clinically significant threshold of 0.1% burden	

PFA, pulsed field ablation; AA, atrial arrhythmia.

## 6. Anesthesia

PV isolation can be performed under general anesthesia (GA) or conscious sedation (CS), depending on patient preference, operator preference, and the expertise and experience at the performing center. Generally, CS is considered less expensive, offers a shorter recovery period from the sedation, requires less time preparing for the anesthesia and a shorter stay in the catheter laboratory after the procedure. In contrast, GA requires a pre-procedural examination of the patient by the anesthesiologist, which may lead to a longer hospitalization, and result in specific complications associated with intubation or ventilation [60]. A single center study comparing CS to GA found no significant difference with regard to safety and efficacy but increased GA time and procedure cost [61]. However, the ablation procedure is considered painful and since patients report different pain thresholds, CS can be ineffective in some patients. In these cases when patients are uncomfortable and tend to move, an electro-anatomical map may shift, and a redo of a map may be required, thus prolonging the time of the procedure [62]. The best strategy for anesthesia remains controversial and no standardized approach for the use of sedation and analgesia is described. Most of the studies comparing CS vs GA were conducted with RFA. These studies demonstrated that in GA, greater catheter stability and signal attenuation is obtained, leading to better accuracy of the mapping system, and thus lower rates of recurrence [63, 64]. Another study confirmed the superiority of GA over CS for catheter stability using a new evaluation method based on the distance traveled by the catheter distal tip per second, and demonstrated less periprocedural complications [65]. Recently, artificial intelligence was employed to analyze the raw data from real-time three-dimensional maps system and evaluate procedural parameters. It showed that GA improves the quality of lesions and the procedural efficiency of PVI [66]. The main reported benefits of GA are patients’ comfort and shorter total RF energy application time and a shorter fluoroscopic time due to complete elimination of interruption of RF energy applications, resulting from increased patient movement and excessive respiratory excursions [62]. Time of the procedure may be further optimised with continuous infusion of fentanyl [67]. Patient discomfort is also an important point to consider, as one randomized trial demonstrated, that patients who undergo ablation under CS are less likely to agree to a repeat procedure compared to those treated under GA [66]. In contrast, there is data, that very high-power short-duration temperature-controlled radiofrequency ablation may reduce ablation times and improve patient tolerability, permitting PV isolation using only benzodiazepine in most of patients without compromising the patient’s pain experience [68].

There is a lack of studies comparing anesthesia methods during CBA, but one single center study demonstrated CS was associated with shorter total electrophysiological laboratory time without a significant effect on the recurrence of atrial arrythmias or complication rates. The authors contend that while the use of GA sometimes may be necessary, it should be used electively [69]. The use of CS during CBA is commonly used in clinical practice. A large meta-analysis of 11 studies with CBA found that CS was used in 8 studies, CS or GA in 1 study, and GA alone in just 1 study [47]. Studies comparing sedation outcomes across established energy modalities have also been conducted. One study reported no differences in sedation-related complications between RFA and CBA, despite the longer procedure times associated with RFA [70].

Data on sedation in PFA remains limited. The “5S Study” demonstrated the safety of sedation during PFA [71]. Similarly, the MANIFEST-PF study reported safe sedation in 82.1% of cases [72]. A recent study comparing sedation in PFA and CBA found that PFA requires higher levels of sedation, particularly analgesics, suggesting greater intraprocedural pain sensation compared to CB ablation [70]. This might be associated with PFA-induced muscle contractions, coughing, and phrenic nerve injury. However, complication rates were similar between the two technologies, indicating that sedation in PFA is as safe as in CB ablation [73]. Further studies comparing the type of anesthesia, especially in PFA, are required.

## 7. Catheter Technologies

Contemporary catheters can be categorized as either circumferential PV isolation tools or point-by-point ablation devices. Historically, the first developed technology was point-by-point RFA. Later, single-shot devices have been developed to streamline the PVI procedure. Studies have documented that the single-shot CBA demonstrates simmilar results to RFA [74]. Second-generation techniques, such as second generation cryoballoon and contact force guided RFA using 3D mapping were developed to increase the efficacy of the PVI [75]. Contact force sensing technology allowing continuous CF monitoring during ablation optimised effective tissue lesion by ensuring optimal contact force between the catheter tip and target tissue, while second generation cryoballoon optimized lesions in various settings of PV anatomies demonstrating reduced time to isolate PV, procedural time, and overall success compared with the first-generation balloons [75, 76]. However, no significant differences regarding safety or efficacy between those two advanced techniques were observed [75].

**Fig. 1.**
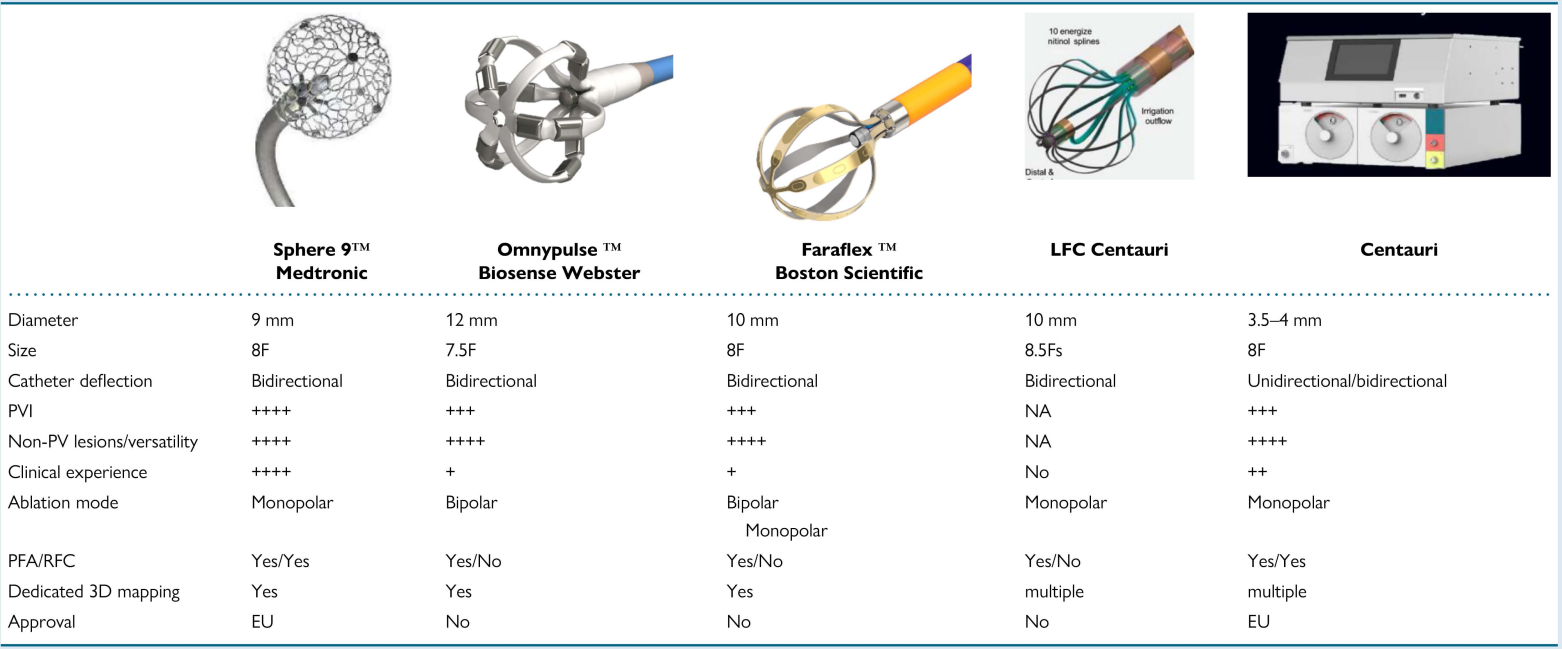
**Overview of focal point-by-point large/intermediate footprint PFA catheters and a specific generator enabling PFA using conventional RF catheters**. (Adapted from Chun *et al*. [79]). RF, radiofrequency; PV, pulmonary vein; RFC, radiofrequency current; the “+” quantity indicates the catheter’s suitability for the specified characteristic.

**Fig. 2.**
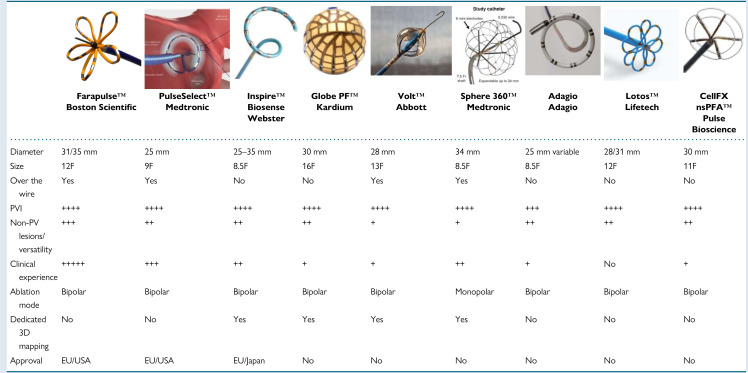
**Overview of contemporary circumferential PVI tools**. (Adapted from Chun *et al*. [79]). 3D, three dimensional; the “+” quantity indicates the catheter’s suitability for the specified characteristic.

Due to recent advancements in PFA technology, numerous new catheters, designs, and generators are currently under development and are being evaluated in pre-clinical and clinical studies. In search of methods for safety and convenience, single-shot PFA catheters have been developed. The first ones introduced into clinical practice were multielectrode array catheters fashioned in either a fixed-loop configuration or as an adjustable pentaspline catheter. Later, a high-fidelity multielectrode variable-loop circular catheter, used with a multichannel PFA generator and dedicated electroanatomic mapping system, was designed [77]. Focal catheters permit flexibility of lesions beyond PVI, and large-area focal, deflectable catheters have been shown to facilitate efficient, point-by-point ablation strategies in clinical studies [78]. Circumferential PVI tools are presented in Fig. 1 (Ref. [79]), focal point-by-point PFA catheters are presented in Fig. 2 (Ref. [79]). Recently a PFA catheter, utilizing a specific generator, that can deliver both energy sources—pulsed field and radiofrequency, was developed to facilitate the creation of contiguous lesion sets. This technology allows the ability to toggle between RFA and PFA. Data from a multicenter study demonstrated that the use of dual energy allows for more efficient procedures, increases durability of lesions, and results in good freedom from paroxysmal and persistent AF [80]. The SmartfIRE trial further confirmed that the dual energy focal contact-force catheter integrated with 3D mapping achieves high first-pass isolation rates and 100% acute procedural success in the treatment of paroxysmal AF [78]. Prespecified 3-month remapping demonstrated significant PVI durability with an acceptable safety profile, reinforcing its efficacy and reliability in this clinical setting [81]. Further advancements in 3D mapping systems are expected in the near future to facilitate the creation of contiguous lesion sets and reduce the risk of gaps. Moreover, PFA may contribute to a better understanding as to whether AF recurrences are due to insufficient mapping or ablation. Such advancements are likely to establish PFA as a cornerstone in the treatment of AF.

The left atrial posterior wall plays a key role in persistent atrial fibrillation due to its embryologic, anatomic, and electrophysiologic properties. In patients with persistent AF, where pulmonary vein isolation alone is often insufficient, it serves as a potential ablation target. However, clinical studies report mixed results regarding the safety and efficacy of posterior wall isolation [82]. Not all catheters are suitable for posterior wall isolation, as their effectiveness depends on design, energy source, and maneuverability. Commonly used options include radiofrequency catheters, cryoballoons, and multi-electrode catheters. Contact-force sensing radiofrequency catheters are frequently used, though achieving durable isolation can be challenging due to the need for precise point-by-point ablation. Cryoballoon catheters are effective for pulmonary vein isolation but may not provide complete posterior wall coverage. More recently, multi-electrode mapping and pulsed field catheters have gained attention for their ability to achieve durable and safe isolation. Some catheters, particularly older-generation radiofrequency catheters, may lack stability, precision, or the ability to create contiguous lesions, making them less suitable for posterior wall isolation.

## 8. Non-Ablation Strategies for Controlling Atrial Fibrillation

Emerging evidence suggests that sodium-glucose cotransporter 2 (SGLT2) inhibitors (SGLT2i) may positively affect arrhythmia-related outcomes, particularly in AF. Post hoc analyses of major SGLT2i trials indicated a reduced incidence of new-onset AF in patients treated with SGLT2i compared to placebo. A meta-analysis of 31 trials involving over 75,000 participants found that SGLT2i reduced the risk of serious AF events [83]. However, the effects of SGLT2i on pre-existing AF are less understood. It is hypothesized that SGLT2i may reduce AF progression and related healthcare visits by improving metabolic function and structural remodeling of the heart. A cohort study of adults with diabetes and AF showed that patients on SGLT2i had lower AF-related healthcare utilization and improved outcomes, including reduced all-cause mortality and heart failure (HF) hospitalizations, compared to those on dipeptidyl peptidase-4 inhibitors (DPP4i). These findings suggest that SGLT2i could be beneficial for managing AF in patients with diabetes [84].

Another promising strategy in managing patients with AF is the “ablate and pace” approach. It was found that in patients with AF and heart failure, atrioventricular junction ablation combined with biventricular pacemaker was more effective than pharmacological rate control in reducing mortality. In a trial of 133 patients with permanent AF and prior HF hospitalizations, ablation combined with a resynchronization therapy reduced all-cause mortality significantly, with 5% mortality at 2 years compared to 21% in the drug group. The benefit was consistent regardless of baseline ejection fraction. Additionally, the combined endpoint of mortality or heart failure hospitalization was also lower in ablation plus resynchronization group [85].

A systematic review and meta-analysis by Mei *et al*. [86] found that algorithms for right ventricular pacing minimization (RVPm) effectively reduce the risk of persistent AF, cardiovascular hospitalization, and heart failure hospitalization in patients requiring anti-bradycardia therapies. These benefits were observed across different algorithm types and in patients with both sick sinus node disease and atrioventricular block. RVPm algorithms successfully reduced pacing below the 20% threshold, minimizing pacing-induced complications. Continuous monitoring via modern pacemakers enables early AF detection, which is a strong predictor of stroke and AF progression. Potential concerns include PR interval prolongation and mode-switch effects, but no significant adverse symptoms or syncope were noted. Subgroup analysis confirmed consistent efficacy across different algorithms and patient groups. Additionally, RVPm algorithms may help extend pacemaker battery life, reducing replacement risks.

These findings support the integration of RVPm algorithms into clinical practice, complementing physiological pacing strategies.

These studies provide valuable insights into alternative strategies that could complement or potentially replace ablation therapies.

## 9. Conclusions

AF represents a significant healthcare challenge, marked by increasing rates of morbidity and mortality. The role of catheter ablation in managing these patients is rapidly increasing. Current clinical guidelines recommend its use as a first-line treatment option in patients with paroxysmal AF (IA class of recommendation) and in selected patients with persistent AF (IIb class of recommendation). The comparison of various ablation techniques reveals distinct advantages relevant to clinical practice. RFA, as the first energy source introduced into clinical practice, has demonstrated higher effectiveness compared to drug therapy. CBA has also established itself as a safe and effective alternative, particularly for symptomatic paroxysmal AF. As the main concern regarding thermal ablation is the lack of selectivity to cardiomyocytes and thus induced complications such as PV stenosis, phrenic nerve palsy, and atrial-esophageal fistula, the PFA has emerged as a promising method, offering noninferior efficacy to conventional thermal techniques while potentially minimizing complications associated with thermal damage. However, it is essential to consider the higher equipment costs of PFA. Thus, while each ablation technique presents unique benefits, the selection of the appropriate method should be tailored to individual patient profiles, treatment objectives, and available resources. Ongoing research will be crucial in further refining optimal strategies for effectively managing AF.

## Availability of Data and Materials

Data sharing is not applicable to this article as no datasets were generated or analyzed during the current study.
